# Overcoming barriers to the implementation of patient-reported outcomes in cancer clinical trials: the PROMOTION Registry

**DOI:** 10.1186/1477-7525-12-86

**Published:** 2014-06-06

**Authors:** Fabio Efficace, Jonathan Rees, Peter Fayers, Andrea Pusic, Martin Taphoorn, Elfriede Greimel, Jaap Reijneveld, Katie Whale, Jane Blazeby

**Affiliations:** 1 Data Center and Health Outcomes Research Unit, Italian Group for Adult Hematologic Diseases (GIMEMA), Via Benevento, 6, 00161 Rome, Italy; 2 School of Social and Community Medicine, University of Bristol, Bristol, UK; 3 Institute of Applied Health Sciences, University of Aberdeen, Aberdeen, UK; 4 Department of Surgery, Memorial Sloan Kettering Cancer Center, New York, USA; 5 VU University Medical Center, Department of Neurology, Amsterdam, The Netherlands; 6 Department of Obstetrics and Gynecology, Medical University of Graz, Graz, Austria; 7 Department of Neurology, VU University Medical Center, Amsterdam, The Netherlands; 8 Centre for Surgical Research, University of Bristol and Division of Surgery, University Hospitals Bristol NHS Foundation Trust, Bristol, UK; 9 Medical Center Haaglanden, Department of Neurology, The Hague, The Netherlands

**Keywords:** Cancer, Patient-reported outcomes, Clinical trials, Quality of life, Clinical decision-making

## Abstract

Every cancer treatment, irrespective of its clinical effectiveness, has an impact on patients’ quality of life (QoL). Even recently developed targeted therapies might have side effects and significantly impact patients’ QoL. Thus, understanding the advantages and disadvantages of different treatments from the patient’s standpoint has become a must in clinical research and is highly valued by major stakeholders. Thousands of cancer patients are enrolled into randomized controlled trials (RCTs) each year and many complete patient-reported outcome (PRO) instruments to obtain patient-centered information as part of the assessment of the overall effectiveness of the new therapy. Some of these RCTs have generated high quality PRO evidence forming the basis for approval (or support to approval) of drugs by the US Food and Drug Administration. However, a consistent strategy to determine the quality of patient centered evidence presented in RCTs has until recently been lacking. One of the fundamental questions when including PROs in clinical research revolves around methodological robustness and consistency of outcome reporting. Cancer patients, physicians and healthcare system stakeholders need to rely on solid information to make the best possible choice regarding treatment. Therefore generating high-quality findings from PRO assessment in cancer trials is of paramount importance. In an effort to improve quality of PRO assessment and reporting in the near future, the Patient-Reported Outcome Measurements Over Time In ONcology (PROMOTION) Registry was developed. The scope of this Registry is to identify, track, analyse, and store information on all cancer RCTs that have included PROs, and assess the quality of their PRO assessments.

## Background

Cancer is now an increasingly common diagnosis worldwide as population longevity increases. Recent data from the International Agency for Cancer research (IARC) indicate approximately 14.1 million new cancer cases in 2012, a rise of more than 10% compared to 2008 (12.7 million cases), and further projections suggest an increase to 19.3 million new cancer cases per year by 2025 [1]. Therefore, understanding how cancer and its treatment impact on each aspect of a patient’s life is critical and remains a major challenge.

Every cancer treatment, irrespective of its clinical effectiveness, has an impact on patients’ quality of life (QoL). Standard treatments have traditionally included chemotherapy, radiotherapy and surgery or a combination of these. In recent years, anticancer-targeted therapies have been developed for some cancers, and very often these greatly contribute to improving both survival and QoL [2,3]. Nonetheless, even targeted therapies might have side effects and significantly impact patients’ QoL [4]. Thus, understanding the advantages and disadvantages (*pro* and *cons*) of different treatments from the patient’s standpoint has become a must in clinical research and is highly valued by major stakeholders [5].

### Rationale and objective

Randomized controlled trials (RCTs), play a key role in cancer research as they provide the best methodology to evaluate treatments and inform health policy and practice. Within RCTs in oncology, it is now commonplace to make assessment of patient-reported outcomes (PROs). Thousands of cancer patients are enrolled into RCTs each year and many complete relevant PRO instruments to obtain patient-centered information as part of the assessment of the overall effectiveness of the new therapy. A number of these RCTs have generated high quality PRO evidence and some of these have also formed the basis for approval (or support to approval) of drugs by the US Food and Drug Administration (FDA) by showing better PROs with a given treatment. Examples of this include mitoxantrone for prostate cancer patients and imatinib for patients with chronic myeloid leukemia [3,6]. However, it should be noted that methodological drawbacks in the design and reporting of PROs have often limited the use of PRO data in approval of drugs [7].

However, a consistent strategy to determine the quality of patient centered evidence presented in RCTs has until recently been lacking, but is vital to clinicians as PRO data from RCTs may directly inform patients and practitioners regarding the effects of a treatment, or may indirectly influence clinical practice by incorporation into relevant guidelines [8]. One of the fundamental questions when including PROs in clinical research revolves around methodological robustness and consistency of outcome reporting. There are a number of well known challenges, for example, to PRO data collection, appropriate timing of assessment, adequate statistical analysis and outcome interpretation [9,10]. In addition, the choice of the PRO questionnaire is also a fundamental aspect to be considered; the questionnaire should be relevant for the specific research question, and with solid psychometric characteristics (e.g., valid, reliable and responsive to health changes). Cancer patients, physicians and healthcare system stakeholders need to rely on solid information to make the best possible choice regarding treatment. Therefore generating high-quality findings from PRO assessment in cancer trials is of paramount importance. Poorly designed or reported PROs assessment is likely to hamper the critical appraisal of results and limit the knowledge transfer from research settings to clinical practice.

The European Organisation for Research and Treatment of Cancer (EORTC) Quality of Life Group (QLG) has pioneered the field of methodological research in this area by developing and validating an international portfolio of measures to be used in RCTs in oncology [11]. The EORTC has led on several published reviews of PRO in RCTs, showing that the application of PROs in many trials was plagued by important methodological drawbacks, limiting the generalizability of the results [12-15]. In response to these findings the EORTC raised the need to establish international standards for QoL assessment in cancer clinical trials [16] and made significant efforts to define basic minimum criteria to guide investigators when reporting HRQOL from cancer RCTs [17]. Further international initiatives have subsequently been launched to increase the quality of PRO assessment in clinical trials, including guidance from the International Society for Quality of Life Research (ISOQOL) [18]. The ISOQoL guidance is of particular value as it formed the basis for the development of the recently issued CONSORT PRO recommendations [19].

In an effort to improve quality of PRO assessment and reporting in the near future, the EORTC QLG recently contributed to devise the Patient-Reported Outcome Measurements Over Time In ONcology (PROMOTION) Registry (http://promotion.gimema.it). This is also co-financed by the Italian Group for Adult Hematologic Diseases (GIMEMA). The broad scope of this Registry is to identify, track, analyse, and store information on all cancer RCTs that have included PROs, and assess the quality of their PRO assessments. This work is being performed using a common set of criteria across all cancer disease sites, thus allowing comparisons across all cancer areas.

### Overview on registry content and type of information included

The database stores information stemming from published RCTs that enrolled at least 50 patients overall, that report PROs, and that have been published since 2004. To date, nearly 600 studies in a wide range of cancer populations have been identified, analyzed and electronically archived. Data on more than 50 different items have been extracted from each eligible study.

This information is categorized into three broad groups, that is: 1) Study characteristics, including summary of clinical and PRO results; 2) PRO methodology, based on the recently published recommendations by ISOQOL [18] and the CONSORT PRO group [19] and 3) Risk of bias assessed using the Cochrane Risk of Bias tool. We acknowledge the evaluation of this latter aspect might be challenging by just reading the publication of study results, and inspection of full study protocol would be valuable. In any case, this tool specifically evaluates the adequacy of sequence generation, allocation concealment, blinding of participant/personnel, incomplete outcome data reporting, blinding of outcome assessment, selective outcome reporting and other possible sources of bias for each RCT [20]. For descriptive purposes, in Table 1 we report a summary of some of the key data collected in the registry (Table 1).

**Table 1:** **Descriptive summary of some key data contained in the PROMOTION Registry (**http://promotion.gimema.it**)**

***Basic Study Characteristics***	Name of Cooperative Group/s leading the study (if any)
	Study location
	Industry support
	Primary endpoint/s
	Difference between treatment arms in the primary endpoint (if any)
	Age of patients
	Gender of patients
	Disease stage
	Overall trial sample size
	PRO sample size
	PRO instrument/s used
	Summary of PRO results
	If statistically significant PRO difference exists, details of the domain/s of interest should be reported (e.g. symptoms only, functional aspects, global quality of life)
	Summary of main clinical (other than PRO) results
***PRO Methodology and Analysis***	PRO identification in the abstract
	Statement of PRO hypothesis and its PRO domain
	Description of the mode of administration of the PRO tool and the methods of collecting data
	Electronic mode of PRO administration
	Description of the rationale for choice of the PRO instrument
	Citation of evidence of PRO instrument validity and reliability
	Description of the intended PRO data collection
	Statement of the status of PRO as either a primary or secondary outcome
	Statement of the magnitude of the effect size (for statistically significant PRO results)
	Description of statistical approaches for dealing with missing data
	Statement of the extent of missing data
	Flow diagram or description of the allocation of participants and those lost to follow-up for PROs specifically
	Statement of the reasons for missing data
	Description of the study patients’ characteristics including baseline PRO scores
	Reporting of PRO outcomes in a graphical format
	Discussion of the limitations of the PRO components of the trial
	Discussion of the limitations of the clinical significance of the PRO findings
	Methodology used to assess clinical significance
	Discussion of the PRO results in the context of the other clinical trial outcomes
***Study Validity***	Selection bias
	Performance bias
	Detection bias
	Attrition bias
	Reporting bias

Abbreviation: PRO (Patient-Reported Outcome).

*Legend:* the following aspects are reported for descriptive purposes only. This is not a comprehensive list of all data contained in the PROMOTION Registry.

### Overview on working methodology

All published RCTs meeting the inclusion criteria are identified by electronic searching of PubMed/Medline, the Cochrane library, PsycINFO and PsycARTICLES. Hand searching of the bibliographies of eligible articles are also performed.

After eligibility of articles for inclusion is confirmed, a minimum of two trained reviewers (who are also study collaborators) independently examine each trial and extract pre-specified information. Every reviewer has a personal password to access the online system to complete the electronic-data extraction form (eDEF), details of which have been reported previously [21]. This approach allows a double blind data entry procedure to ensure accuracy and validity of data entry into the Registry. Discrepancies in the independent evaluations are recorded and a discrepancy form automatically generated by the system. Discrepancies are discussed by the two reviewers and resolved by them with a third reviewer reconciling any continuing discrepancies. After the review team reach consensus for all items the final eDEF for the RCT is locked, validated and imported in the Registry for research use.

### Future work and value to the international community

The methodological challenge of incorporating PROs into cancer RCTs is frequently cited as a major limiting factor for their use. However, through the PROMOTION Registry data we have begun to show that PRO methodological quality has improved over time in RCTs in prostate disease, suggesting that the methodological challenges related to PRO logistics, design and analyses are no longer insurmountable [21].

Work continues within the PROMOTION Registry team to determine if this is also the case for other cancer sites, and to identify key areas where PRO methodology remains poor in cancer RCTs and needs to be addressed to facilitate the translation of PRO findings from research to clinical practice.

The PROMOTION Registry (http://promotion.gimema.it) is now an extensive source of information for all investigators involved in PROs related cancer research and will continue to be regularly updated. Data and information contained in the registry can be used to address specific clinical or more methodological research questions. We welcome research proposals from other investigators and research groups (not only from GIMEMA and the EORTC QLG) to collaborate with the international PROMOTION team in the effort to drive PROs into clinical practice and better inform healthcare decisions.

## Competing interests

All authors declare that they have no competing interest.
